# Harnessing the Noncanonical Keap1-Nrf2 Pathway for Human Cytomegalovirus Control

**DOI:** 10.1128/jvi.00160-23

**Published:** 2023-03-20

**Authors:** Ayan K. Ghosh, Yu-Pin Su, Michael Forman, Robert F. Keyes, Brian C. Smith, Xin Hu, Marc Ferrer, Ravit Arav-Boger

**Affiliations:** a Department of Pediatrics, Division of Infectious Disease, Medical College of Wisconsin, Milwaukee, Wisconsin, USA; b Department of Pediatrics, Division of Infectious Disease, Johns Hopkins University School of Medicine, Baltimore, Maryland, USA; c Department of Pathology, Johns Hopkins University School of Medicine, Baltimore, Maryland, USA; d Department of Biochemistry, Program in Chemical Biology, Medical College of Wisconsin, Milwaukee, Wisconsin, USA; e National Center for Advancing Translational Sciences (NCATS), National Institutes of Health, Rockville, Maryland, USA; The University of Arizona

**Keywords:** human cytomegalovirus, noncanonical Keap1-Nrf2 pathway, SQSTM1/p62, antioxidant response element, ARP101, p62-Keap1-Nrf2

## Abstract

Host-derived cellular pathways can provide an unfavorable environment for virus replication. These pathways have been a subject of interest for herpesviruses, including the betaherpesvirus human cytomegalovirus (HCMV). Here, we demonstrate that a compound, ARP101, induces the noncanonical sequestosome 1 (SQSTM1)/p62-Keap1-Nrf2 pathway for HCMV suppression. ARP101 increased the levels of both LC3 II and SQSTM1/p62 and induced phosphorylation of p62 at the C-terminal domain, resulting in its increased affinity for Keap1. ARP101 treatment resulted in Nrf2 stabilization and translocation into the nucleus, binding to specific promoter sites and transcription of antioxidant enzymes under the antioxidant response element (ARE), and HCMV suppression. Knockdown of Nrf2 recovered HCMV replication following ARP101 treatment, indicating the role of the Keap1-Nrf2 axis in HCMV inhibition by ARP101. SQSTM1/p62 phosphorylation was not modulated by the mTOR kinase or casein kinase 1 or 2, indicating ARP101 engages other kinases. Together, the data uncover a novel antiviral strategy for SQSTM1/p62 through the noncanonical Keap1-Nrf2 axis. This pathway could be further exploited, including the identification of the responsible kinases, to define the biological events during HCMV replication.

**IMPORTANCE** Antiviral treatment for human cytomegalovirus (HCMV) is limited and suffers from the selection of drug-resistant viruses. Several cellular pathways have been shown to modulate HCMV replication. The autophagy receptor sequestosome 1 (SQSTM1)/p62 has been reported to interact with several HCMV proteins, particularly with components of HCMV capsid, suggesting it plays a role in viral replication. Here, we report on a new and unexpected role for SQSTM1/p62, in HCMV suppression. Using a small-molecule probe, ARP101, we show SQSTM1/p62 phosphorylation at its C terminus domain initiates the noncanonical Keap1-Nrf2 axis, leading to transcription of genes under the antioxidant response element, resulting in HCMV inhibition *in vitro.* Our study highlights the dynamic nature of SQSTM1/p62 during HCMV infection and how its phosphorylation activates a new pathway that can be exploited for antiviral intervention.

## INTRODUCTION

Infection with human cytomegalovirus (HCMV), a member of the herpesvirus family, is common. Seroprevalence rates increase with age, reaching 90% at 80 years (1). HCMV establishes lifelong persistent infection, and individuals typically remain asymptomatic. In immunocompromised hosts, HCMV causes significant morbidity and mortality (2, 3). HCMV-seropositive HIV-infected patients progress more rapidly to AIDS than those who are CMV seronegative (4, 5).

HCMV is the most common congenitally acquired infection worldwide (6). It is the leading infectious cause of hearing loss, mental retardation, and central nervous system damage in children. The annual societal cost of supporting children with congenital HCMV approaches $3 billion, based on data from 1991 (7).

Most systemic anti-HCMV drugs target the viral DNA polymerase. Their use results in toxicities to the bone marrow (ganciclovir [GCV]) and kidneys (foscarnet and cidofovir) and the emergence of resistant viruses (8–11). These serious issues drive the search for new agents and therapeutic strategies for HCMV. Letermovir, a terminase inhibitor, was recently approved for CMV prophylaxis after hematopoietic stem cell transplantation (12, 13), and resistance is already reported (14). The UL97 kinase inhibitor maribavir (MBV) received FDA approval in 2021 for adults and children (12 years of age and older) with refractory HCMV disease. The limited availability of drugs for HCMV therapy, the side effects associated with existing drugs, and the emergence of resistant viral mutants during therapy generated significant interest in identifying host-directed anti-HCMV therapeutics (9, 10, 15–18).

We screened the LOPAC library of 1280 pharmacologically active compounds and identified several hits which may inhibit HCMV using host-directed pathways (19). We selected hit ARP101 (a matrix metalloproteinase-2 inhibitor) for further studies, based on its reported ability to inhibit cancer cells’ growth via autophagy induction (20–23). ARP101 also inhibited α-melanocyte-stimulating hormone (α-MSH)-stimulated melanogenesis by regulating autophagy in mouse fibroblasts (24). Induction of autophagy has been reported to restrict HCMV replication (25). Digitoxin, a cardiac glycoside, and trehalose, a nontoxic naturally occurring disaccharide, induce autophagy to inhibit HCMV replication *in vitro*. Digitoxin appears to induce autophagy at early stages through AMP-activated protein kinase (AMPK) activation, while trehalose increases the levels of Rab7, a protein required for lysosomal biogenesis and fusion (26, 27).

We show that ARP101 exerts its anti-HCMV activity not via autophagy induction but rather through a different pathway that is activated by the autophagy receptor/adaptor, SQSTM1/p62 (here abbreviated as p62). p62 is a central molecule that acts as a hub for major catabolic pathways including autophagy (selective and nonselective) and the ubiquitin-proteasomal system (28, 29).

The nuclear factor erythroid 2-related factor 2 (Nrf2) is a master regulator of oxidative stress. Under homeostatic conditions, Nrf2 levels and its activation are governed by Kelch-like ECH-associated protein 1 (Keap1) (30). During oxidative or electrophilic challenge, Nrf2 is activated and induces the expression of its target genes, involved in cellular protection. This mechanism of Nrf2 activation is known as the canonical activation of the pathway (31). Recently, a noncanonical mechanism has been described, where the activation of Nrf2 is carried out by other proteins, p62, DPP3 (dipeptidyl peptidase III), WTX (Wilms’ tumor gene on the X chromosome), PALB2 (protein partner and localizer of BRCA2), p21, and BRCA1 (breast cancer type 1 susceptibility protein), which selectively bind to Keap1 with greater affinity and dislocate Nrf2 in the process (32–36).

p62 was reported to colocalize with HCMV capsid and tegument proteins: major capsid protein and smallest capsid protein (MCP/sCP) in the nucleus and the inner tegument protein pp150 in the cytoplasmic viral assembly compartment (37). p62 accumulation was also observed in HCMV-infected cells, and it was incorporated into purified extracellular viral particles, exhibiting its role in virion maturation (38). The C-terminal region of p62 consists of three major domains: an LC3-interacting region (LIR), a Kelch-like ECH-associated protein 1 (Keap1)-interacting region (KIR), and a ubiquitin-associated domain (UBA) containing multiple phosphorylation sites (29, 39). Among several phosphorylation sites present in the C-terminal region, Ser403 and Ser407 (S403, UBA domain) and Ser349 (S349, KIR domain) play an important role in selective autophagy (40, 41). Phosphorylation of Ser349 at the KIR domain also triggers the noncanonical Keap1-Nrf2 pathway by displacing Nrf2 from the interaction with Keap1 due to enhanced affinity of p62 for Keap1 (42, 43).

We report here on HCMV inhibition by ARP101 via induction of the noncanonical p62-Keap1-Nrf2 pathway during the early-late stage of viral replication. Our studies reveal a novel pathway for HCMV inhibition in which ARP101 treatment phosphorylates p62 at its KIR and UBA domains, activating the Keap1-Nrf2 pathway, resulting in transcription of stress-responsive genes under the antioxidant response element (ARE). Our study illuminates a novel mechanism of ARP101, involving the adaptor protein p62 as an effector of an antiviral signaling cascade.

## RESULTS

### ARP101 inhibits HCMV replication.

**FIG 1:**
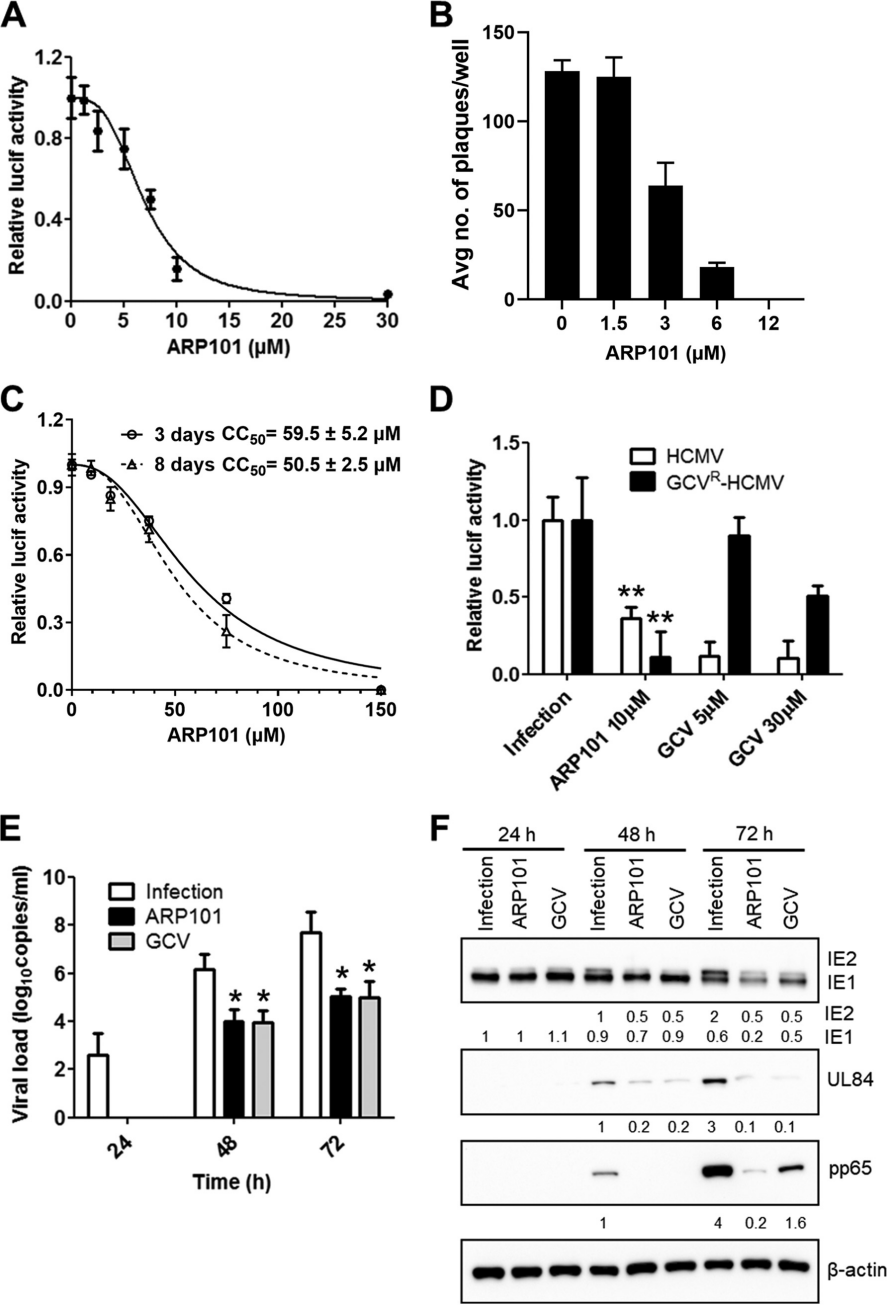
HCMV inhibition by ARP101. (A) A luciferase-based reporter assay was performed at 72 hpi on HFFs infected with the pp28-luciferase recombinant HCMV-Towne (MOI of 1 PFU/cell), followed by treatment with the indicated concentrations of ARP101. (B) HCMV-TB40 (200 PFU/well) was used to infect HFFs (1 × 10^6^ cells in a 24-well plate), followed by treatment with the indicated concentrations of ARP101. On day 8 postinfection, plaques were enumerated. (C) HFFs were treated with the indicated concentrations of ARP101 for 72 h and 8 days, and a luminescence-based cell viability assay was performed to determine the 50% cellular viability (CC_50_). (D) The activity of ARP101 (10 μM) against pp28-luciferase ganciclovir (GCV)-resistant HCMV-Towne (GCV^R^ HCMV) was measured by luciferase assay at 72 hpi. (E) HFFs were infected with HCMV-Towne (MOI of 0.1) for 24, 48, and 72 h and treated with ARP101 (10 μM) or GCV (5 μM). Infected cells were lysed, DNA was isolated, and viral load was measured by a US17 quantitative real-time PCR. (F) HFFs were infected with HCMV-Towne (MOI of 1) and treated with ARP101 (10 μM) or GCV (5 μM). Infected cells were lysed at 24, 48, and 72 hpi, lysates were run on 12% SDS-PAGE gels, and immunoblot assays were performed to detect IE1/2 (immediate early), UL84 (early late), and pp65 (late) antigens. Numbers presented below each panel of immunoblots represent the relative band intensities of each blot. Relative luciferase activity was calculated by dividing the luciferase units obtained at a given drug concentration by the luciferase units obtained following treatment with the vehicle control, dimethyl sulfoxide. All experiments were performed in triplicates, and represented values are mean ± SD.

Autophagy induction is a suggested mechanism for HCMV inhibition (26, 27). ARP101 reportedly induces autophagy in several cancer cell types and inhibits melanogenesis in melanocytes by regulating autophagy (20–24). Here, we investigated its activity and mechanism of HCMV inhibition. Human foreskin fibroblasts (HFFs) were infected with pp28-luciferase recombinant HCMV-Towne (multiplicity of infection [MOI], 1 PFU/cell) and treated with ARP101 at concentrations ranging from 1 to 30 μM. The EC_50_ (effective concentration resulting in 50% luciferase inhibition) of ARP101 was 6.5 ± 0.23 μM (Fig. 1A). A plaque assay using HCMV-TB40 (200 PFU/well) revealed an EC_50_ of 2.9 ± 0.1 μM (Fig. 1B). The CC_50_ (50% cellular toxicity) of ARP101 measured in noninfected HFFs at 72 h and 8 days was 59.5 ± 5.2 μM and 50.5 ± 2.5 μM, respectively (Fig. 1C). The slope of ARP101 was 3.1 ± 0.7, and the selectivity index (SI) for HCMV-Towne and HCMV-TB40 was 9.15 ± 0.86 and 17.4 ± 1, respectively. ARP101 also inhibited ganciclovir (GCV)-resistant pp28-luciferase recombinant Towne (Fig. 1D). Additionally, ARP101 reduced cell-associated viral DNA by ≥1 log_10_ at 48 and 72 h postinfection (hpi). GCV was used as a positive control (Fig. 1E). To understand the effect of ARP101 on the expression of viral proteins, an immunoblot assay was performed using lysates of infected and treated cells at different time points following infection. ARP101 effectively inhibited IE1/2, UL84, and pp65 expression at 48 and 72 hpi (Fig. 1F). GCV (5 μM) reduced IE2, UL84, and pp65 at 48 and 72 hpi. β-Actin was probed as a loading control (Fig. 1F).

**FIG 2:**
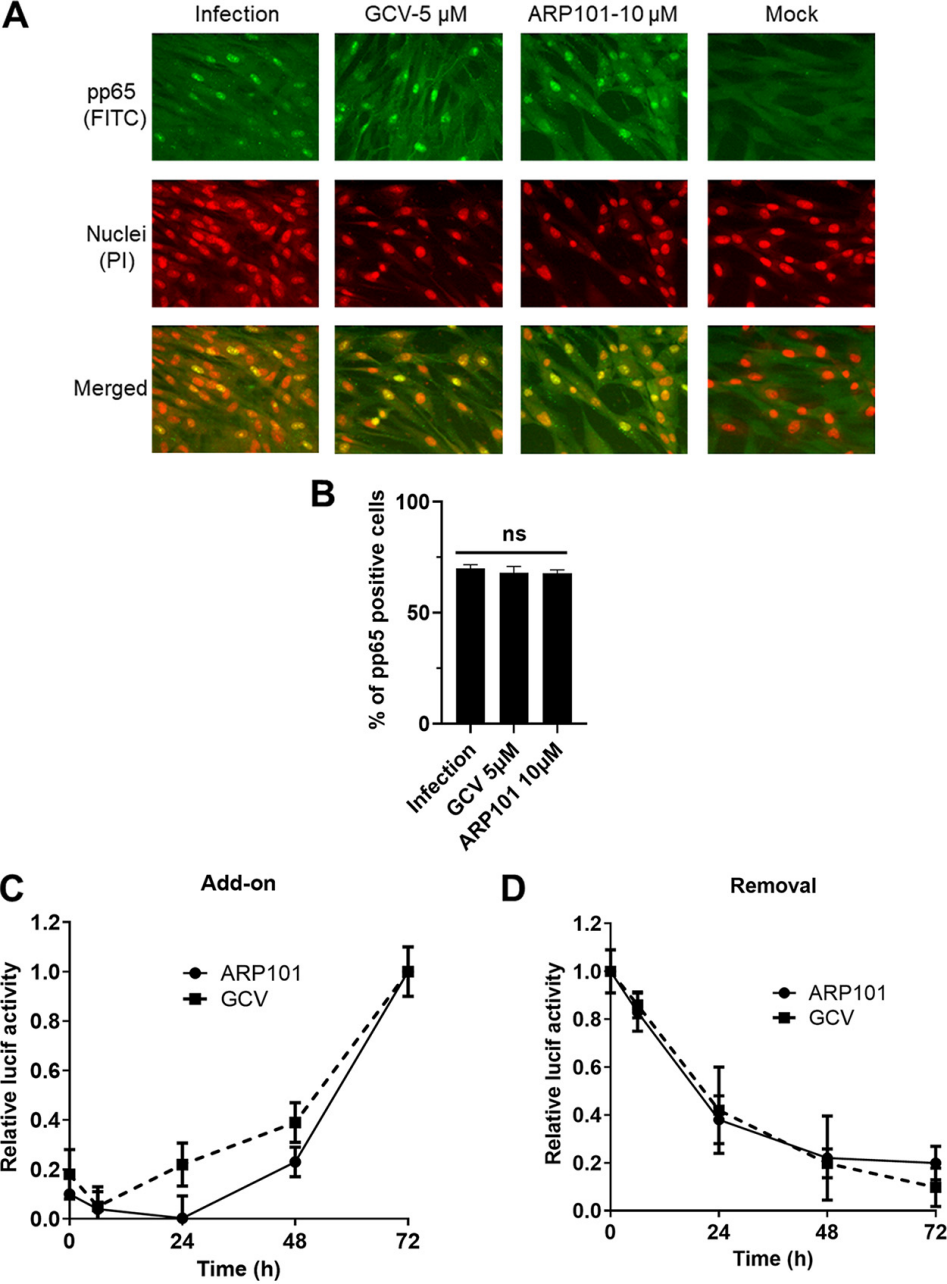
Timing of activity of ARP101. (A) An immunofluorescence assay was performed to test for HCMV entry. HFFs were pretreated with dimethyl sulfoxide (mock), ARP101, or GCV for 24 h and then infected with HCMV-Towne (MOI of 1). (B) Graph depicting percentage of pp65-positive cells in the microscopic data presented in panel A. (C and D) A luciferase-based reporter assay was performed to determine the timing of activity of ARP101. HFFs were infected with pp28-luciferase recombinant Towne, and ARP101 (10 μM) was either added to or removed from infected HFFs at 6, 24, 48, and 72 h postinfection. GCV (5 μM) was used as a control for timing of activity. Relative luciferase activity was calculated by dividing the luciferase units obtained at a given drug concentration by the luciferase units obtained following treatment with the vehicle control, dimethyl sulfoxide. All experiments were performed in triplicates, and represented values are mean ± SD.

We next investigated the timing of ARP101 activity during infection. An indirect fluorescence assay was performed to determine if ARP101 inhibits HCMV entry. HFFs were pretreated with ARP101 or GCV for 24 h, followed by infection with HCMV-Towne (MOI of 1 PFU/cell). Similarly to GCV, ARP101 did not inhibit virus entry (Fig. 2A and B). In all panels, the pp65 signal colocalized with the nuclear propidium iodide (PI) staining, indicating ARP101 does not inhibit HCMV entry into HFFs. Add-on and removal assays were next performed. ARP101 was either added or removed from infected cells at 6, 24, 48, and 72 hpi. Based on luciferase activity, ARP101 was ineffective when added after 48 hpi and completed its anti-HCMV activity when removed at 48 hpi, suggesting its maximal time of activity is at the early-late time of infection, similar to GCV (Fig. 2C and D).

### ARP101 induces both LC3 II and p62 during infection.

**FIG 3:**
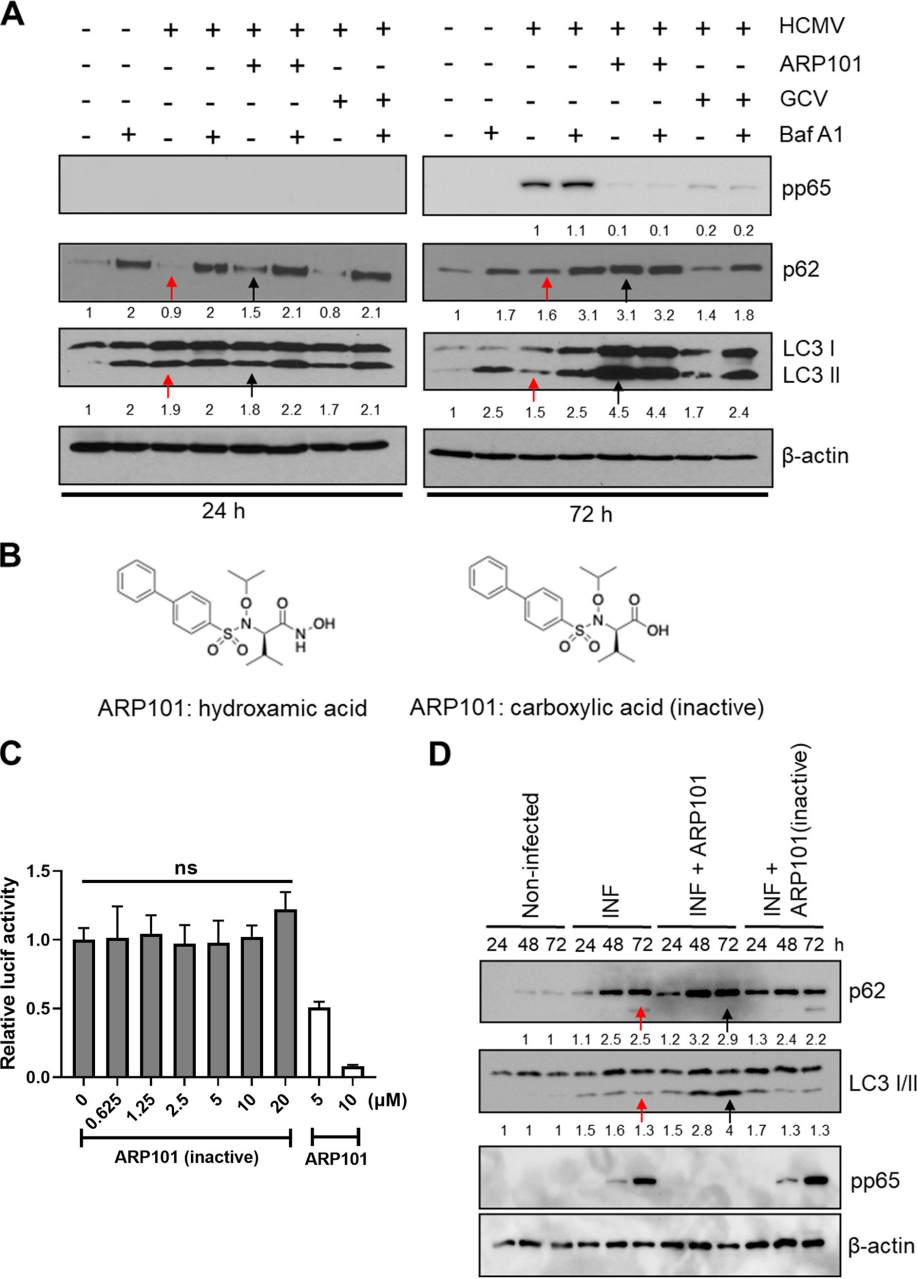
ARP101 induces both p62 and LC3 II in HCMV-infected HFFs. (A) HFFs were infected with HCMV-Towne (MOI of 1) and treated with ARP101 (10 μM) for 24 and 72 h. Bafilomycin A1 (Baf A1, 50 nM) was added to the respective conditions 3 h before harvesting. Following Baf A1 treatment, cells were lysed, and an immunoblot assay was performed for LC3 I/II and p62. Red arrows indicate HCMV-infected HFF samples, and black arrows indicate ARP101-treated HCMV-infected HFF samples. HCMV pp65 and β-actin were probed as infection and loading controls, respectively. (B) Structure of active ARP101 (having the hydroxamic acid functional group) and inactive ARP101 (carboxylic acid replaces the hydroxamic acid functional group). (C) HCMV inhibition by ARP101 hydroxamic acid and the carboxylic acid (inactive) was measured at 72 hpi with pp28-luciferase recombinant Towne (MOI of 1). (D) Whole-cell lysates from infected HFFs were analyzed by immunoblotting for p62, LC3 I/II, and viral pp65 following treatment with the active and inactive ARP101 (10 μM) at the indicated time points. Red arrows indicate HCMV-infected HFF samples, and black arrows indicate ARP101-treated HCMV-infected HFF samples at 72 h postinfection. The experiments were performed in triplicates, and the best representative data are presented. INF, infected with HCMV-Towne.

Since ARP101 was reported to induce autophagy in cancer cell lines (22–24), we tested its effects on the autophagic flux. HFFs were infected with HCMV-Towne (MOI of 1), and LC3 I/II level was measured at 24 and 72 hpi. The initial virus-induced LC3 II level at 24 hpi was decreased at 72 hpi (1→1.9→1.5), whereas the expression of the autophagy receptor p62 was increased (1→0.9→1.6 [Fig. 3A, red arrows]). In contrast, ARP101 induced both LC3 II (1.8→4.5) and p62 (1.5→3.1) levels at 72 hpi (Fig. 3A, black arrows). GCV (5 μM), as expected, did not alter either LC3 II or p62 level compared to infected controls. Using bafilomycin A1 (Baf A1; an inhibitor of autophagosome and lysosome fusion) ARP101-treated samples displayed a similar increase in LC3 II and p62 levels (Fig. 3A). HCMV pp65 level was reduced with ARP101 at 72 hpi and was unchanged by bafilomycin A1 (Fig. 3A). An inactive version of ARP101 (carboxylic acid; -COOH), lacking the functional hydroxamic acid group -CONHOH (Fig. 3B), did not reduce pp28-luciferase activity or the level of viral pp65, did not induce LC3 II in HCMV-infected cells, and showed similar levels of p62 as infected cells at 48 and 72 hpi (Fig. 3C and D).

### ARP101-mediated virus inhibition does not involve the classical autophagy pathway.

**FIG 4:**
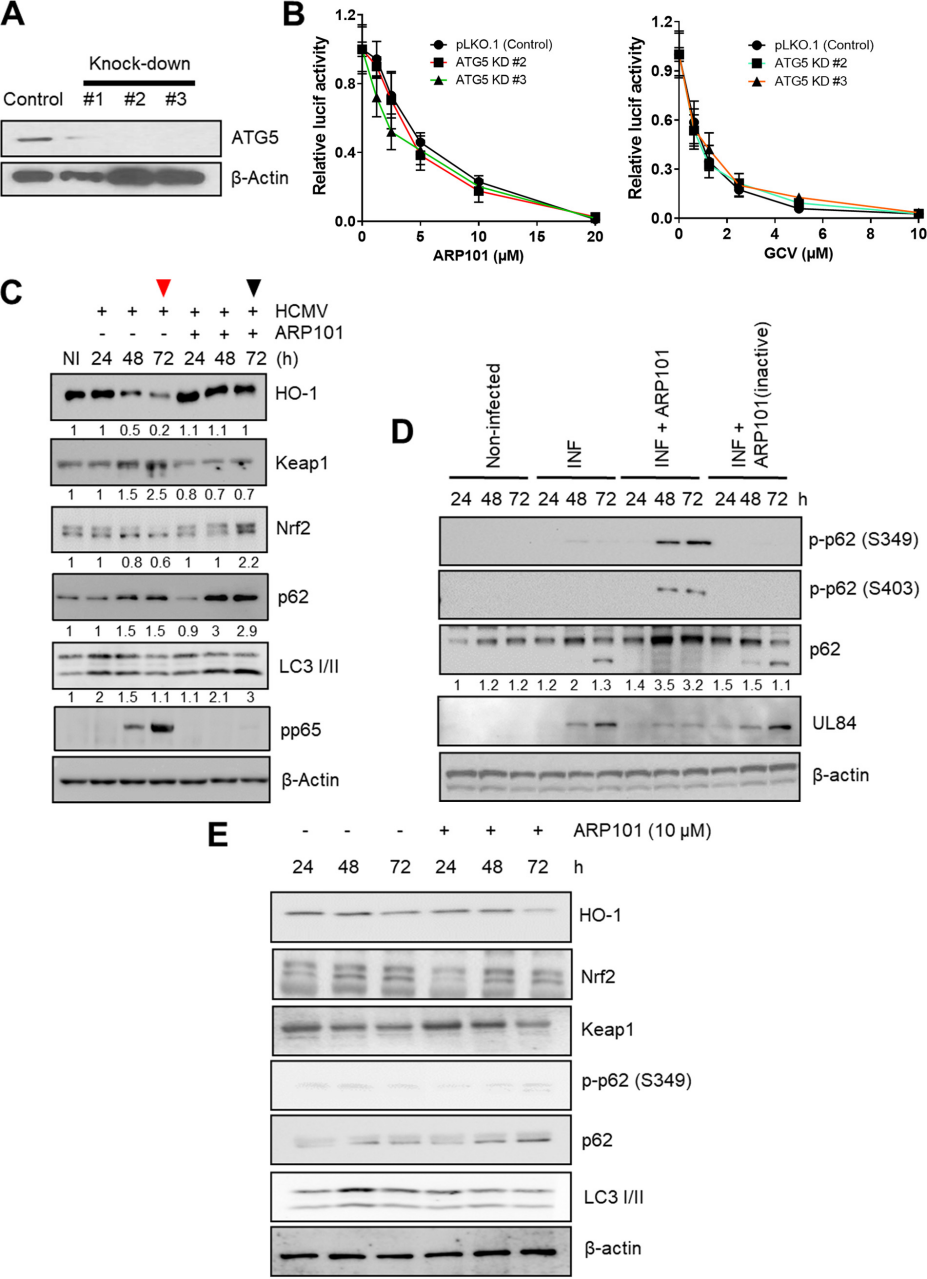
ARP101 induces phosphorylation of p62 at the KIR and UBA domains and increases expression of Nrf2 and heme oxygenase-1 (HO-1). (A) ATG5 knockdown (KD) by lentiviral transduction in HFFs. Cells expressing pLKO.1 (vector control) and plasmids containing ATG5 shRNA were grown to confluence following puromycin selection and lysed in cell lysis buffer. ATG5 expression in pLKO.1 control and ATG5 KD HFFs (#1, #2, and #3) was analyzed by immunoblotting. (B) A luciferase-based reporter assay was performed with ATG5 KD (#2 and #3) and pLKO.1 control HFFs infected with HCMV-Towne (MOI of 1) and treated with the indicated concentrations of ARP101 and GCV. The values are represented as the mean ± SD from two independent experiments. (C) HFFs were infected with HCMV-Towne (MOI of 1) and treated with ARP101 (10 μM) for 24, 48, and 72 h. Cells were lysed posttreatment, and equal amounts of proteins were analyzed on 12% SDS-PAGE gels. Immunoblot assays were performed for HO-1, Keap1, Nrf2, p62, and LC3 I/II. Viral pp65 and β-actin were probed for infection and loading controls, respectively. The red arrowhead indicates HCMV-infected HFF samples, and the black arrowhead indicates ARP101-treated HCMV-infected HFF samples at 72 hpi. (D) HCMV-infected cells (MOI of 1) were lysed posttreatment with active and inactive ARP101 (10 μM). Equal amounts of lysates were analyzed on 12% SDS-PAGE gels, and an immunoblot assay was performed for p-p62 (S349), p-p62 (S403), p62, viral UL84, and β-actin. The experiment was repeated in triplicate, and representative images are presented. (E) HFFs were treated with ARP101 (10 μM) and harvested at 24, 48, and 72 h posttreatment for analysis of levels of HO-1, Nrf2, Keap1, p-p62 (S349), p62, and LC3 I/II by Western blotting. β-Actin was probed as an internal control. NI, noninfected; INF, infected with HCMV-Towne.

To understand the involvement of classical autophagy in ARP101 activity, ATG5, a major autophagy regulator, was knocked down (KD) in HFFs by lentiviral transduction. Vector control (pLKO.1) and three distinct ATG5 KD lentiviruses were used to transduce HFFs and were selected by puromycin treatment for at least two passages following transduction. KD of ATG5 protein level was observed in transduced cells (Fig. 4A). ARP101 maintained its anti-HCMV activity in both ATG5 KD and control cells, suggesting classical autophagy was not required for HCMV inhibition by ARP101 (Fig. 4B). GCV had similar antiviral activities in ATG5 KD and control cells (Fig. 4B). The data suggest that ARP101-mediated induction of LC3 II and p62 uses a novel mechanism of action in HCMV-infected cells.

### ARP101 induces the noncanonical p62-Keap1-Nrf2 pathway.

LC3 II and p62 are inversely related in HCMV-infected HFFs; decreased autophagy late during infection correlates with p62 accumulation (Fig. 3A) (44). Since in infected ARP101-treated cells, both p62 and LC3 II were induced, we asked how the level of p62 was maintained despite increased LC3 II level. p62 is transcribed as part of the cellular antioxidant response element (ARE) after successful translocation and binding of Nrf2 to the ARE promoter (45). Therefore, we probed the p62-Keap1-Nrf2 pathway proteins in HCMV-infected cells and after ARP101 treatment. Heme oxygenase-1 (HO-1) level was decreased at 48 and 72 hpi (1→0.5→0.2), but HO-1 was highly expressed following ARP101 treatment in HCMV-infected cells (Fig. 4C, red and black arrowheads). The data in HCMV-infected cells corroborate previous reports of HO-1 mRNA and protein induction until 48 hpi (46, 47).

At 48 and 72 hpi ARP101 treatment increased the level of p62 despite the accumulation of LC3 II. Keap1 expression was reduced compared to infected controls (Fig. 4C, lane with black arrowhead), while Nrf2 expression increased at 72 hpi with ARP101 treatment. The expression of pp65 was used as a marker of late HCMV infection, and β-actin was probed for loading control (Fig. 4C).

To investigate the mechanism of activation of the Keap1-Nrf2 pathway during HCMV infection, p62 phosphorylation was measured at Ser349 (S349; located in the Keap1-interacting domain [KIR]) and Ser403 (S403; located at the ubiquitin-associated domain [UBA]). Both sites are key effectors of p62 binding to Keap1 and other designated cargos, resulting in noncanonical activation of the Keap1-Nrf2 pathway (43, 48). ARP101 strongly induced p62 phosphorylation at Ser349 and Ser403 at 48 and 72 hpi (Fig. 4D) but not in noninfected cells (Fig. 4E). p62 phosphorylation was noticed only with ARP101 (Fig. 4D and Fig. 3B; hydroxamic acid) but not with the inactive version of ARP101 (Fig. 4D and Fig. 3B; carboxylic acid), suggesting specific activation of the noncanonical p62-Keap1-Nrf2 pathway by ARP101 during HCMV infection.

In summary, ARP101-mediated p62 phosphorylation at Ser349 and Ser403 results in decreased Keap1 levels, stabilizing Nrf2. The latter is expected to bind the ARE promoter sites, supported by the induction of HO-1 at 48 and 72 hpi in ARP101-treated infected cells (Fig. 4C and D).

### ARP101 treatment results in nuclear translocation of Nrf2 followed by ARE activation and reduced Keap1 levels in the cytoplasm.

**FIG 5:**
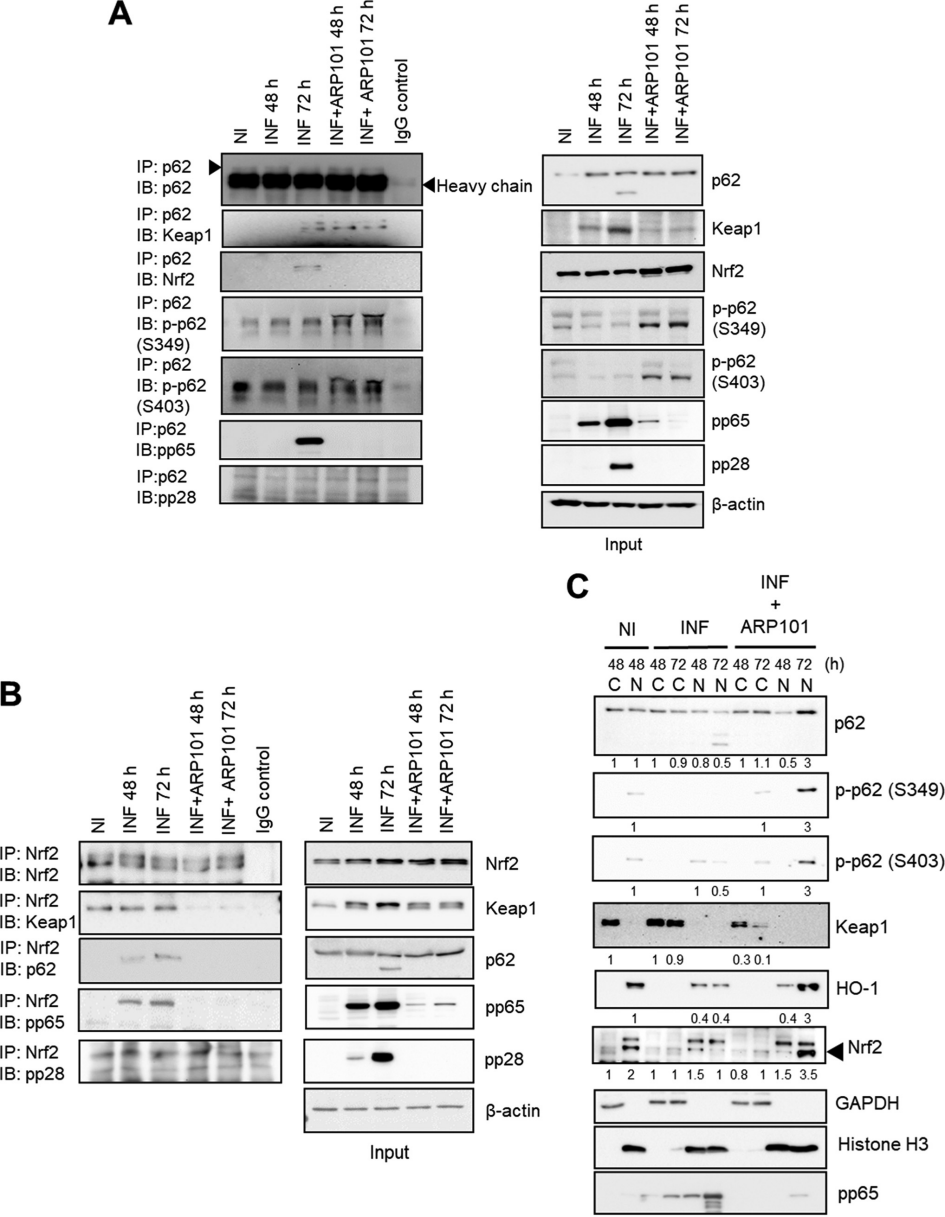
ARP101 releases Nrf2 from the p62-Keap1-Nrf2 complex, resulting in nuclear translocation. (A) Immunoprecipitation (IP) of p62 was performed in HCMV-infected (MOI of 1) ARP101 (10 μM)-treated cells at 48 and 72 hpi. Keap1, Nrf2, p-p62 (S349), and p-p62 (S403) were probed to measure the affinity of p62 for Keap1 and its effect on Nrf2 release from Keap1. Viral pp65 and pp28 were probed for analysis of the interaction of p62 with viral proteins. The right panel shows the profile of different proteins in the soluble fraction of the lysates (input), which was used for IP. IB, immunoblot. (B) Reverse IP of Nrf2 was performed in HCMV-infected ARP101-treated cells at 48 and 72 hpi. Keap1 and p62 were probed to measure the status of the p62-Keap1-Nrf2 complex. The right panel shows the input used for the IP. (C) HFFs were infected with HCMV-Towne (MOI of 1) and treated with ARP101 (10 μM) for 48 and 72 h. Cells were fractionated into cytoplasmic (C) and nuclear (N) fractions, concentrated by acetone precipitation, and quantified. Equal amounts of proteins were analyzed on 12% SDS-PAGE gels, and immunoblot assays were performed to measure the level of p62, p-p62 (Ser349), p-p62 (S403), Keap1, Nrf2, and HO-1. Histone H3 and GAPDH were probed as nuclear (N) and cytoplasmic (C) controls, respectively. Viral pp65 was probed as an infection control. The experiments were performed thrice, and representative images are presented. NI, noninfected; INF, infected with HCMV-Towne. IgG control experiments were conducted with INF cell lysates.

We next investigated the changes in p62-Keap1-Nrf2 interaction following infection and ARP101 treatment. Immunoprecipitation (IP) of p62 in infected cells revealed that at 72 h p62 interacted with both Keap1 and Nrf2, but with ARP101 treatment, phosphorylation of p62 (S349 and S403) resulted in displacement of Nrf2 from the complex (Fig. 5A). At 48 hpi, IP with p62 antibody did not pull down Keap1, but with ARP101 treatment, as p62 was phosphorylated at S349 and S403, p62 associated with Keap1 (Fig. 5A). Interestingly, pp65 (but not pp28, a true late viral protein) also precipitated with Keap1 and Nrf2 in infected cells at 72 hpi. A reverse IP with Nrf2 antibody confirmed that the Nrf2-Keap1 complex was associated with p62 at both 48 and 72 hpi along with the viral pp65. In contrast, ARP101 disrupted the Nrf2-Keap1 complex secondary to enhanced affinity of p-p62 (S349 and S403) for Keap1 (Fig. 5B).

**FIG 6:**
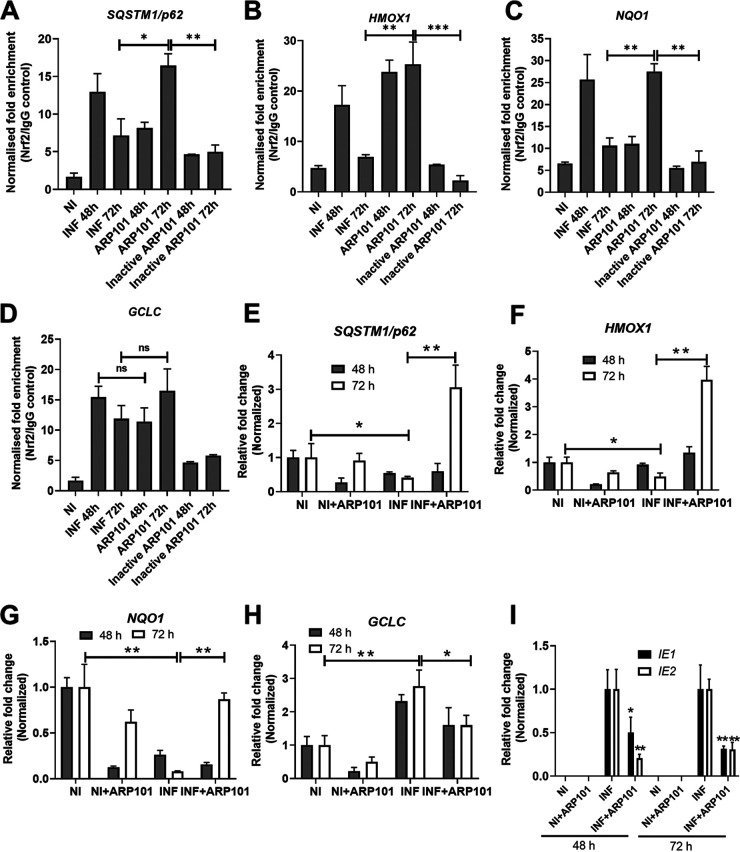
ARP101 induces binding of Nrf2 to specific promoters and activation of the antioxidant response element (ARE). (A to D) Binding of Nrf2 to several ARE promoters was analyzed by ChIP assay. HFFs were infected with HCMV-Towne (MOI of1) and treated with ARP101 or inactive ARP101 (10 μM) for 48 and 72 h. (E to H) Quantitative PCRs were performed for *SQSTM1/p62* (E), *HMOX1* (F), *NQO1* (G), and *GCLC* (H) transcribed upon ARE activation in infected HFFs treated/untreated with ARP101 at 48 and 72 hpi. (I) Viral IE1 and IE2 expression was analyzed by quantitative PCR for infection control in the same samples from panels E to H. The experiments were performed thrice, and data from a single experiment with three technical replicates are presented. NI, noninfected; INF, infected with HCMV-Towne.

The cellular localization of p62-Keap1-Nrf2 was next analyzed during infection and ARP101 treatment at 48 and 72 hpi. Nrf2 localization to the nucleus was induced after ARP101 treatment at 72 hpi, likely ensuring ARE activation (Fig. 5C). Keap1 was localized in the cytosol, and its levels were maintained at 48 and 72 hpi but reduced in ARP101-treated HCMV-infected cells (Fig. 5C). ARP101-induced p62 phosphorylation at Ser349 and Ser403 was mostly nuclear and reduced the nuclear p62 degradation at 72 hpi (disappearance of lower-molecular-weight bands seen under the infected-only condition, Fig. 5C). Compared to HCMV-infected cells, total p62 increased in both the cytoplasmic and nuclear fractions following ARP101 treatment at 72 hpi (Fig. 5C). The increased HO-1 level induced by ARP101 was predominantly nuclear at 72 hpi (Fig. 5C). Glyceraldehyde-3-phosphate dehydrogenase (GAPDH) and histone H3 were probed as controls for cytoplasmic and nuclear fractions, respectively. HCMV-pp65 was probed in all samples and was distributed mostly in the nucleus and partly in the cytoplasm (Fig. 5C). A chromatin immunoprecipitation (ChIP) assay was performed to measure the direct effects of ARP101 treatment on nuclear translocation of Nrf2 in HCMV-infected cells. At 72 hpi ARP101 significantly enhanced the binding of Nrf2 to the specific promoter regions of the genes transcribed under the ARE, including *SQSTM1/p62*, *HMOX1*, and *NQO1* (Fig. 6A to C). There was no significant change in Nrf2 binding to specific promoter region of *GCLC* between ARP101-treated and untreated infected cells (Fig. 6D) at 48 or 72 hpi. The specificity of the effect of ARP101 on Nrf2 binding to specific promoters of ARE-regulated genes was confirmed by use of inactive ARP101 (Fig. 3B), where binding was significantly lower than that of the active ARP101 at 72 hpi (Fig. 6A to D).

ARE activation following nuclear translocation and binding of Nrf2 to specific promoter regions by ARP101 treatment was analyzed by quantitative PCR of *NQO1*, *GCLC*, *HMOX1*, and *SQSTM1/p62* at 48 and 72 hpi. The expression of *SQSTM1/p62* and *HMOX1* was significantly upregulated by ARP101 treatment at 72 hpi, but not in noninfected cells, signifying ARE induction following Nrf2 nuclear accumulation. ARP101 treatment also induced transcription of *NQO1*, comparable to the noninfected cellular level, and reduced independent upregulation of *GCLC* by HCMV (Fig. 6E to H). Viral IE1/2 was reduced at 48 and 72 hpi by ARP101 (Fig. 6I). In conclusion, in infected ARP101-treated cells, p62 interacts with Keap1 with increased affinity compared to infected-only cells, resulting in nuclear accumulation of Nrf2 and binding to specific promoters of ARE-regulated genes, ultimately enacting ARE activation. Altogether, ARP101 treatment may result in a feedback loop via the noncanonical Keap1-Nrf2 signaling pathway, which is responsible for increased levels of p62 despite increased LC3 II levels later during infection.

### ARP101 treatment fails to activate the ARE in Nrf2 KD cells.

**FIG 7:**
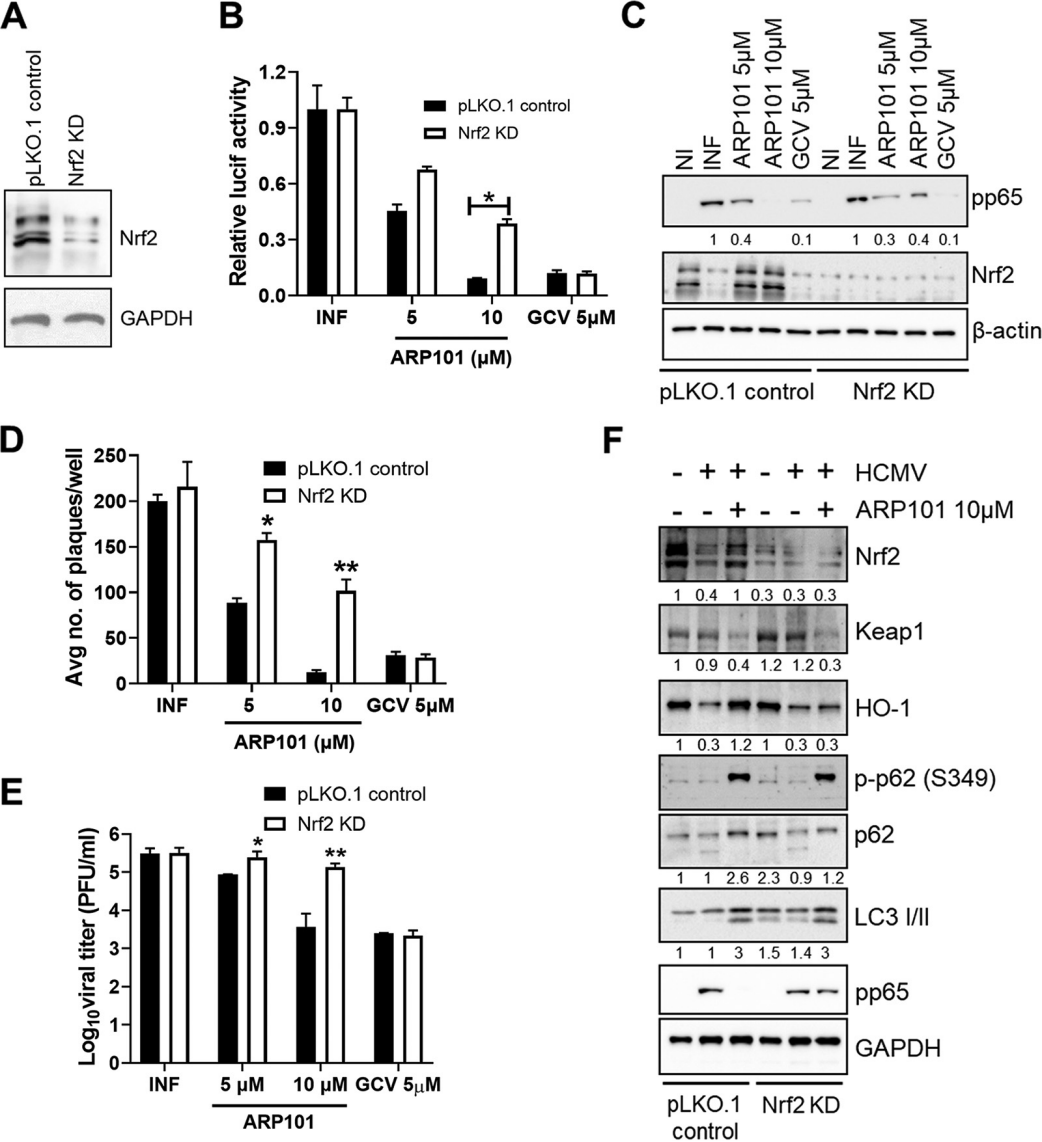
ARP101 fails to activate the ARE in Nrf2 KD cells and does not inhibit HCMV replication. (A) Nrf2 knockdown efficiency was analyzed by immunoblotting in the lysates of lentivirus-transduced HFF cells following puromycin selection. GAPDH was probed as a loading control. (B) A luciferase-based reporter assay was performed with infected (MOI of 1) Nrf2 KD and pLKO.1 control cells treated with the indicated concentrations of ARP101 and GCV. (C) Lysates from panel B were analyzed by Western blotting for Nrf2, viral pp65, and β-actin. (D) A plaque reduction assay was performed using Nrf2 KD and pLKO.1 control HFFs by infecting cells with HCMV-TB40 (200 PFU/well, used to infect 1 × 10^6^ cells in a 24-well plate) and treating them with ARP101 (5 and 10 μM) and GCV (5 μM). (E) Nrf2 KD and pLKO.1 cells were infected with HCMV-TB40 (MOI of 1 PFU/cell), and supernatants were harvested after 120 h followed by titration using plaque assay. (F) Nrf2 KD and pLKO.1 control cells were infected with HCMV-Towne (MOI of 1) and treated with ARP101 (10 μM), and lysates were prepared after 72 hpi. Levels of Nrf2, Keap1, p-p62 (S349), p62, HO-1, and LC3 I/II were measured by Western blotting. Viral pp65 and GAPDH were probed as infection and internal controls, respectively. Experiments were performed thrice, and the best representative images are depicted. NI, noninfected; INF, infected with HCMV-Towne.

To understand the role of nuclear Nrf2 translocation and consequential activation of ARE in the anti-HCMV activity of ARP101, Nrf2 KD HFFs were generated using lentivirus transduction (Fig. 7A). With 10 μM ARP101 treatment, there was an ~3-fold recovery of HCMV replication (pp28 luciferase Towne; MOI of 1 PFU/cell) in Nrf2 KD cells compared to control cells (Fig. 7B). Similarly, when lysates from the same experiment were analyzed by immunoblotting, levels of viral pp65 were recovered by >3-fold in the Nrf2 KD cells compared to control cells (Fig. 7C, ARP101, 10 μM). To confirm these observations, a plaque assay was performed using Nrf2 KD and control cells, showing significantly increased plaque numbers in Nrf2 KD cells compared to control cells at both 5 and 10 μM ARP101 treatment (Fig. 7D). Nrf2 KD also resulted in higher viral titer upon ARP101 treatment (10 μM) at 120 hpi (≥1 log fold) compared to control cells (Fig. 7E). To elucidate the underlying mechanism of improved HCMV replication in Nrf2 KD cells following ARP101 treatment, an immunoblot assay of the Keap1-Nrf2 pathway was performed with the lysates harvested 72 hpi from Nrf2 KD and control cells (Fig. 7F). Decreased levels of both HO-1 and p62 were observed in infected ARP101-treated Nrf2 KD cells compared to control cells, indicating impairment of ARE activation. LC3 II accumulations following treatment with ARP101 were similar in infected Nrf2 KD cells and control cells, suggesting uncoupling of LC3 II from Nrf2. Interestingly, levels of p-p62 (Ser349) and Keap1 were similar in the two cell types with ARP101 treatment, indicative of the involvement of independent kinases in the phosphorylation of p62 which modulates Keap1 level (Fig. 7F). Summarized, ARP101 treatment uses Nrf2 as an effector of the noncanonical p62-Keap1-Nrf2 pathway to inhibit HCMV replication in HFFs.

### Reduction of Keap1 level by ARP101 is p62 dependent.

**FIG 8:**
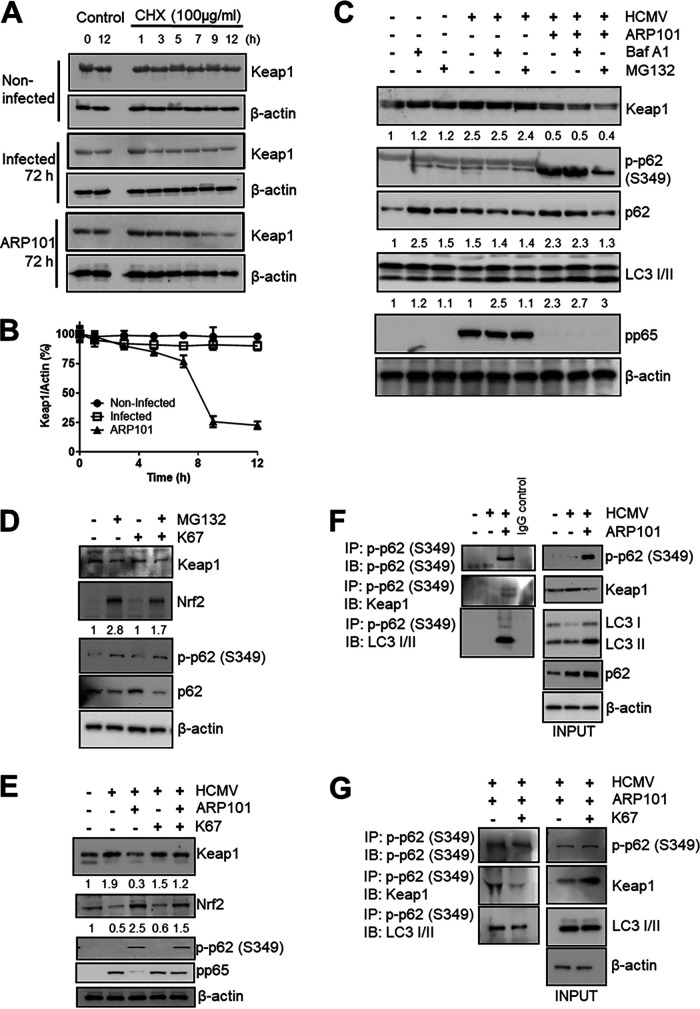
Keap1 is degraded through a p62-mediated process induced by ARP101 in HCMV-infected cells. (A) A cycloheximide chase assay was performed at 72 hpi, and expression of Keap1 was analyzed by immunoblotting. (B) Graphical representation of Keap1 levels measured at 72 hpi in panel A. The level of Keap1 was normalized to β-actin at the indicated time points after the addition of cycloheximide. (C) The levels of Keap1, along with several other proteins, at 72 hpi following ARP101 (10 μM) treatment were analyzed by Western blotting. Bafilomycin A1 (Baf A1; 50 nM) was added 3 h before harvesting at 72 hpi, whereas MG132 (10 μM) was added 8 h before harvesting at 72 hpi. β-Actin was probed as an internal control. (D) HFFs were treated with MG132 (10 μM), K67 (25 μM), and their combination for 18 h, and lysates were prepared for analysis of Keap1, Nrf2, p-p62 (S349), and p62 by immunoblotting. (E) HFFs were infected with HCMV-Towne (MOI of 1 PFU/cell) for 72 h and treated with ARP101 (10 μM), K67 (25 μM), and their combination. Lysates were analyzed by immunoblotting for Keap1, Nrf2, p-p62 (S349), viral pp65, and β-actin, respectively. (F) Immunoprecipitation of p-p62 (S349) followed by detection of Keap1 and LC3 II using noninfected, infected, and ARP101-treated cellular lysates harvested at 72 hpi. (G) IP of p-p62 (S349) followed by detection of Keap1 and LC3 II using HCMV-infected HFFs (MOI of 1 PFU/cell) treated with ARP101 or ARP101 plus K67 and harvested at 72 h postinfection. Experiments were performed thrice, and representative data are presented.

The ARP101-mediated decrease of cytoplasmic Keap1 level at 72 hpi was investigated by a cycloheximide (CHX) chase assay (Fig. 8A). The half-life of Keap1 was decreased at 72 hpi following treatment with ARP101, indicating ARP101-induced degradation of Keap1 at later stages of infection (Fig. 8A and B). To investigate the mode of Keap1 degradation by ARP101, bafilomycin A1 (autophagy inhibitor) and MG132 (inhibitor of proteasomal degradation) were used. ARP101 treatment for 72 h reduced the level of Keap1 compared to infected controls (Fig. 8C). Cotreatment with ARP101 and Baf A1 or with ARP101 and MG132 did not affect LC3 II levels compared to ARP101 alone and did not recover Keap1 levels. These results indicate that neither classical autophagy nor proteasomal degradation was involved in the decreased level of Keap1 (Fig. 8C). To further understand the mechanism of reduced Keap1 level in infected ARP101-treated cells, we used a combination of ARP101 and K67 [an inhibitor of the interaction of p-p62 (Ser349) and Keap1, resulting in Nrf2 degradation]. The effect of K67 on Nrf2 destabilization was first analyzed in noninfected HFFs where phosphorylation of p62 at S349 was induced with MG132. The level of Nrf2 was reduced by the MG132 and K67 combination compared to MG132 alone (Fig. 8D). In infected cells Keap1 level was recovered when ARP101 was used in combination with K67, and viral pp65 level was also restored, suggesting the decrease in Keap1 level is dependent on p-p62 interaction, which is important for HCMV inhibition by ARP101 (Fig. 8E). An IP using p-p62 (S349) antibody showed that p-p62 (S349) associates Keap1 with LC3 II (a key component of autophagosome), resulting in its decreased levels after ARP101 treatment in HCMV-infected cells (Fig. 8F). Additionally, when the IP was performed in the presence of ARP101 and K67, the interaction of p-p62 (S349) with Keap1 was hindered, while only a minimal effect on the association with LC3 II was observed, indicating that p-p62 (S349) acts as the pivot for recruiting Keap1 to the complex (Fig. 8G). In summary, p-p62 (Ser349) interacts with increased affinity with Keap1 following ARP101 treatment of infected cells and propels its degradation via the p62-Keap1-Nrf2 circuit, reducing its half-life in the HCMV-infected cells.

### ARP101-induced p62 phosphorylation at Ser349/403 is not modulated by mTOR kinase or casein kinase 1 or 2.

**FIG 9:**
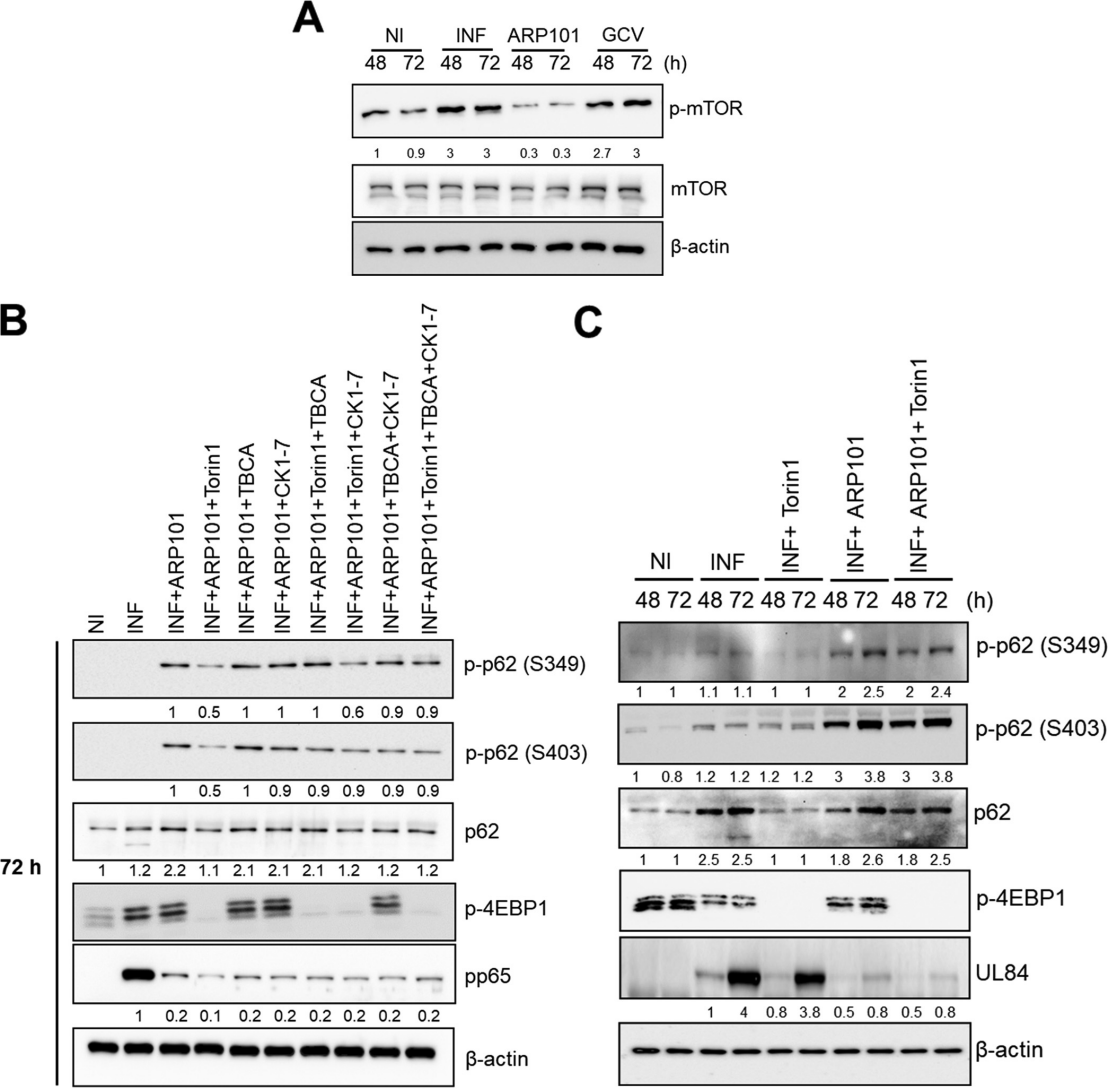
ARP101-induced p62 phosphorylation at S349/S403 is not modulated by the mTOR kinase or casein kinases 1 and 2 in HCMV-infected HFFs. HFFs were infected with HCMV-Towne (MOI of 1 PFU/cell) and treated with ARP101 (10 μM) for 24, 48, and 72 h. Torin-1 (250 nM) was added at 8 h (in panel B) or 24 h (in panel C) before harvesting the cells at 48 or 72 hpi, respectively. TBCA (30 μM), an inhibitor of casein kinase 2, and CK1-7 (50 μM), an inhibitor of casein kinase 1, were also added 8 h before harvesting at 72 hpi. (A) p-mTOR (S2448) and mTOR were analyzed by immunoblotting at the indicated time points. (B) Immunoblot analysis of indicated proteins at 72 hpi under various experimental conditions where kinase inhibitors including Torin-1 were added 8 h before harvesting and ARP101 was added immediately after infection. (C) p-p62 (S349), p-p62 (S403), p62, and p-4EBP1 were analyzed at 48 and 72 hpi following Torin-1 and ARP101 treatments, as mentioned above. Torin-1 was added 24 h before harvesting at indicated time points, and ARP101 was added immediately after infection. Viral pp65/UL84 and β-actin were probed for infection and loading controls, respectively. The experiments were repeated twice, and representative images are presented. NI, noninfected; INF, infected with HCMV-Towne.

The mTOR kinase and casein kinases 1 and 2 were reported to phosphorylate p62 at the Keap1-interacting domain (Ser349 in humans and Ser351 in mice) and Ser403 (43, 49). To test the involvement of mTOR kinase and casein kinases 1 and 2 in ARP101-induced phosphorylation of Ser349 or Ser403 during infection, we used a combination of Torin-1 (selective inhibitor of mTORC1 and mTORC2), TBCA (tetrabromocinnamic acid; selective inhibitor of casein kinase 2), CK1-7 (selective inhibitor of casein kinase 1), and ARP101 in HCMV-infected cells. The level of p-mTOR was first measured in ARP101-treated HCMV-infected HFFs at 48 and 72 hpi (Fig. 9A). ARP101 reduced p-mTOR levels compared to infected controls at 48 and 72 hpi, while GCV treatment did not. This observation suggested that mTOR kinase may play an indirect role in ARP101-induced p62 phosphorylation at Ser349 or Ser403 for HCMV inhibition (Fig. 9A). Cotreatment with ARP101 and Torin-1 (the latter added 8 h before harvesting) reduced p-p62 at both Ser349 and Ser403, but ARP101 plus TBCA or ARP101 plus CK1-7 did not (Fig. 9B). However, in the cotreatment with ARP101 and Torin-1 (the latter added 24 h before harvesting), despite reduced 4EBP1 phosphorylation by Torin-1 (the p-mTOR substrate) at both 48 and 72 hpi, p62 phosphorylation at Ser349 or Ser403 was unchanged among ARP101 alone or under the cotreatment conditions (Fig. 9C). Compared to ARP101 alone, levels of viral pp65 or UL84 (Fig. 9B and C) were unchanged in all the cotreatments of ARP101 plus kinase inhibitors compared to ARP101 alone. Therefore, as the initial decrease in levels of p-p62 (S349/S403) caused by ARP101 and Torin-1 cotreatment (8 h) was recovered at the longer cotreatment duration (24 h), despite clear mTOR inhibition by Torin-1, it can be inferred that independent kinases are involved in p62 phosphorylation at S349/S4030 (Fig. 9B and C). Together, these data indicate the involvement of other kinases in activating the noncanonical p62-Keap1-Nrf2 pathway by ARP101, involving p62 phosphorylation at its C-terminal domain in HCMV-infected HFFs.

## DISCUSSION

We describe a novel mechanism for HCMV inhibition with ARP101 as a probe, by facilitating the phosphorylation of p62 and activating the noncanonical Keap1-Nrf2 pathway. Based on reports of ARP101 inducing autophagy in cancer cells (21–23), the identification of small-molecule modulators of autophagy as potential therapeutic drugs (50, 51), and the role of autophagy in HCMV suppression, our original hypothesis was that ARP101-mediated autophagy may result in HCMV suppression. However, ARP101 treatment revealed a unique pattern in which both LC3 II and p62 were induced later during infection (Fig. 3), and ARP101-induced LC3 II formation was independent of ATG5 (Fig. 4), suggesting the classical autophagy pathway was not required for ARP101 activity. ARP101 facilitated p62 phosphorylation at Ser403 and Ser349, along with a concomitant increase of total p62, indicating a p62-mediated pathway is responsible for HCMV inhibition. Phosphorylation of p62 at its C-terminal region (Ser349, -403, and -407) plays distinct roles, including noncanonical activation of the Keap1-Nrf2 axis and selective autophagy (40–43). For example, phosphorylation of p62 at Ser351 (mouse homolog of Ser349) followed by assembly of p62 on microbes like Salmonella has been described as mechanism of xenophagy, involving noncanonical Keap1-Nrf2 activation (40). There are limited studies on the modulation of the Keap1-Nrf2 pathway during HCMV infection and its role in restricting HCMV replication. In an earlier report, HCMV infection induced Nrf2, which was primarily cytoplasmic with limited translocation into the nucleus, along with a distinct pattern of transcription of Nrf2-induced ARE genes, *HMOX1*, *GCLC*, and *NQO1.* Our data corroborate a prior study showing *GCLC* is upregulated by HCMV at both mRNA and protein levels with concomitant increase in glutathione (GSH) levels. *HMOX1* is induced until 48 hpi, after which its expression returns to basal level, whereas *NQO1* is downregulated starting from 24 hpi (47). We further show here that ARP101 increased *HMOX1* and *NQO1* and decreased *GCLC* mRNA compared to infected cells, possibly representing an indirect effect of HCMV inhibition by ARP101. Lee et al. also observed that Nrf2 played a role in cell viability during infection under oxidative stress but not under normal culture conditions (46). Nrf2 activation has also been associated with an antiviral program and repression of herpes simplex virus 1 replication (52, 53). In our findings, total Nrf2 levels were decreased during the late stage of infection (72 hpi) but stabilized with ARP101 (Fig. 4). Keap1-Nrf2 and p62, along with viral pp65, remained in a complex in infected cells, but ARP101-mediated p62 phosphorylation at Ser349 and Ser403 disrupted the Keap1-Nrf2-p62 complex and promoted Nrf2 translocation into the nucleus, activating ARE gene expression (Fig. 4 to 6). Interestingly, p62 was detected in both the nuclear and cytosolic fractions in HCMV-infected cells, in agreement with earlier reports of p62 entrapment within the major capsid proteins inside the nucleus as well as in the perinuclear viral assembly compartments (Fig. 5), indicative of a role of p62 in viral morphogenesis (37). ARP101-induced p62 phosphorylation at Ser349 and Ser403 could provide an antiviral strategy by the noncanonical Keap1-Nrf2 pathway establishing a feedback loop for regeneration of p62. This is supported by the observation in ARP101-treated Nrf2 KD cells where HO-1 levels were decreased along with p62 and the viral pp65 was restored (Fig. 7).

Keap1 stability plays an important role in the p62-mediated activation of the noncanonical Keap1-Nrf2 pathway and xenophagy (54). Our data show that the Keap1-Nrf2-p62 complex stabilized the half-life of Keap1 in infected cells, but ARP101 treatment reduced the half-life of Keap1 and propelled Keap1 degradation (Fig. 5 and 8). The interaction between p-p62 (Ser349) and Keap1 was the driving force for the degradation, thereby stabilizing Nrf2 and resulting in Nrf2 nuclear translocation and ARE activation (Fig. 5, 6, and 8). Interestingly, a recent study reported that Keap1 is sequestered into p62 gels, which serve as a pivot for autophagosome formation, resulting in Nrf2 activation (55). Although earlier reports suggested Keap1 could be degraded through autophagy (56), our data suggest that p62 plays an important role in Keap1 degradation, linked with inhibition of viral replication. Our data corroborate another study that suggested bafilomycin A1 does not affect Keap1 stabilization, the mechanism of which remains to be determined (Fig. 8) (56). Thus, agents affecting stability of Keap1 could provide a novel mechanism in activation and translocation of Nrf2, by either the canonical or the noncanonical pathway for effective viral inhibition.

Though p-mTOR is induced during HCMV infection, studies have suggested that mTOR activity was not required at the late stage of virus replication (57). Also, the localization of mTOR in the perinuclear viral assembly compartment, along with other autophagy proteins, raised questions regarding the function of mTOR during late stages of virus replication (37, 38, 58). Decreased p-mTOR levels in ARP101-treated whole-cell lysates (Fig. 9) and earlier reports on the involvement of mTOR and casein kinases 1 and 2 in p62 phosphorylation (43, 49) led us to investigate whether ARP101-driven p62 phosphorylation at Ser349/403 was mediated by mTOR or casein kinases. The initial decrease of p-p62 (Ser349 or S403) was replenished later during the cotreatment with ARP101 and Torin-1 and did not have a differential effect on viral replication (Fig. 9). There was no effect on p62 phosphorylation following the cotreatment with ARP101 and any casein kinase inhibitors (Fig. 9). These observations indicate that p62 phosphorylation in both cytosolic and nuclear compartments may be governed by other cytoplasmic/nuclear kinases. The initial decrease of p-p62 (S349/S403) could also be a consequence of autophagy induction by Torin-1, later restored by the feedback loop created by the activation of ARE genes by ARP101 (Fig. 6 and 7). Thus, identification of the novel kinases involved in the phosphorylation of p62 at Ser349 and Ser403 will better define the mechanism of viral inhibition by ARP101 and the trigger of noncanonical activation of the Keap1-Nrf2 pathway.

**FIG 10:**
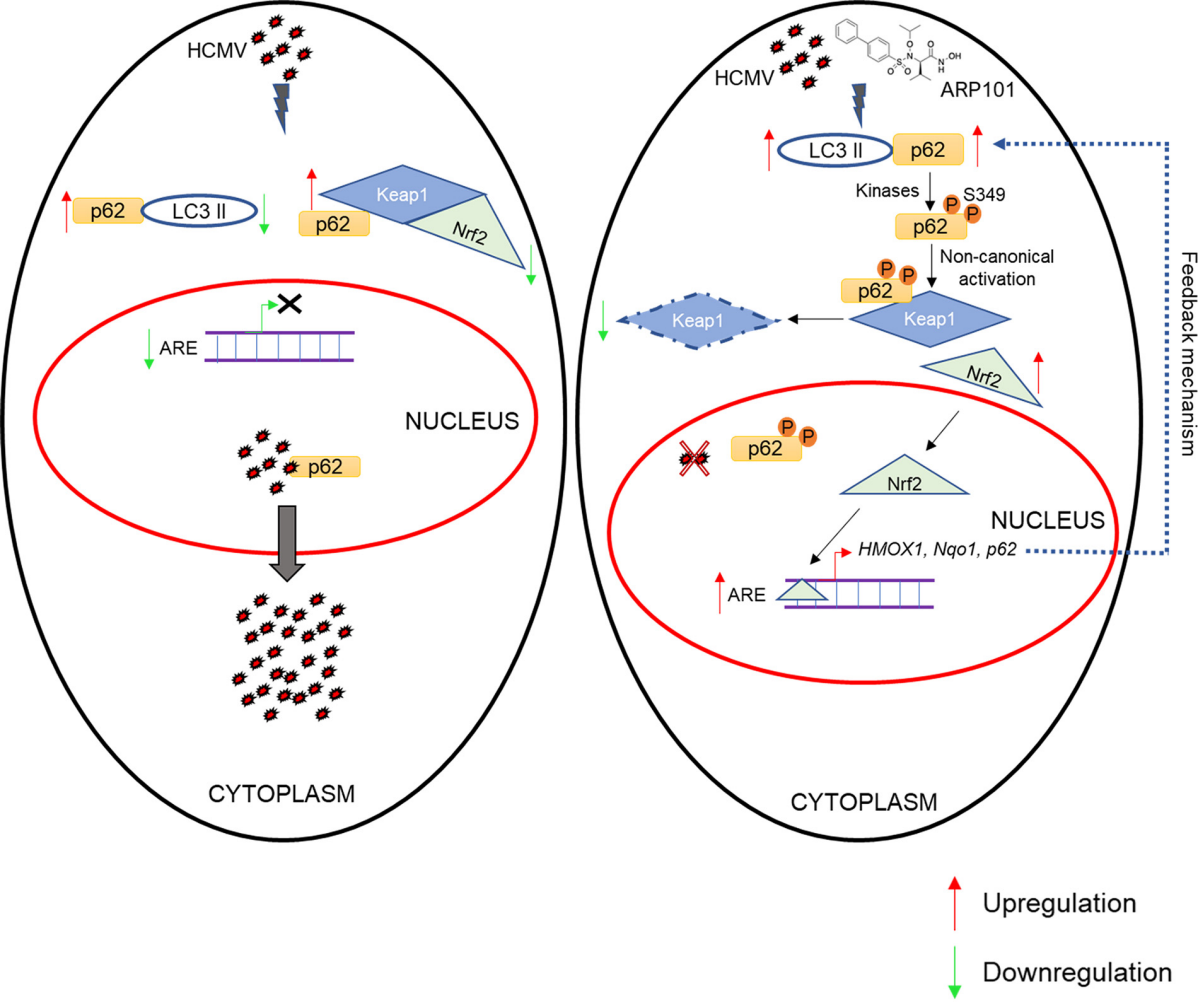
Model depicting the mechanism of anti-HCMV activity of ARP101—induction of the noncanonical p62-Keap1-Nrf2 pathway, resulting in transcription of the antioxidant response element (ARE). (Left) HCMV does not interfere with p62-Keap-Nrf2 interaction and deactivates the ARE for successful replication. (Right) ARP101 induces p62 phosphorylation at Ser349 and Ser403 during infection, resulting in enhanced affinity of p62 for Keap1, and Nrf2 stabilization, which in turn translocates into the nucleus to bind to the specific promoter regions and activate the transcription of genes under the ARE. In the process, Keap1 associated with phosphorylated p62 undergoes degradation via association with LC3 II. Taken together, ARP101 generates a continuous supply of p62 through a feedback loop by the activation of ARE.

Our study identifies a unique participation of p62 in the activation of the noncanonical Keap1-Nrf2 pathway under the influence of ARP101, ultimately inhibiting HCMV replication (Fig. 10). p62 can independently shuttle between the cytoplasm and nucleus (59); its phosphorylation, effect on shuttling, and the changes in the antiviral mechanism of ARP101 will be further investigated in future studies. The specific domains of SQSTM1/p62, its interaction with viral proteins such as pp65, and the effect of phosphorylation on these interactions remain to be investigated.

The activation of Nrf2 has shown promise in chronic diseases of the lung and liver; autoimmune, neurodegenerative, and metabolic disorders; and cancer initiation (60). Recent studies also highlight that Nrf2 agonists 4-octyl-itaconate (4-OI) and the clinically approved dimethyl fumarate (DMF) induce a cellular antiviral program that potently inhibits replication of SARS-CoV-2 across cell lines (61). This led to an interest in developing Nrf2 activators as well as inhibitors of its interaction with Keap1. These compounds are at different stages of development and could provide specific probes to better define HCMV-host cell interaction and their effects on HCMV replication in chronic diseases.

## MATERIALS AND METHODS

### Compounds.

The following compounds were purchased from Sigma-Aldrich (St. Louis, MO, USA), and the indicated concentrations were used throughout the study if not specifically mentioned: ARP101 (10 μM), ganciclovir (GCV; 5 μM), MG132 (10 μM), bafilomycin A1 (50 nM), K67 (25 μM), and cycloheximide (CHX; 100 μg/mL). Torin-1 (250 nM), TBCA (tetrabromocinnamic acid; 30 μM), and CK1-7 (50 μM) were purchased from Cayman Chemical Company (Ann Arbor, MI, USA).

### Synthesis of inactive ARP101 and purity analysis.

**FIG 11:**
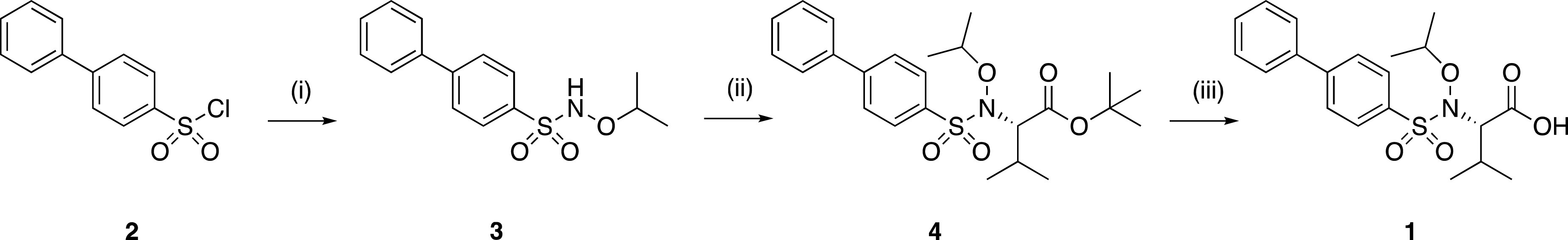
Synthesis of the inactive ARP101 analog compound 1. Reagents and conditions: (i) *O*-isopropylhydroxylamine hydrochloride, *N*-methylpyrrolidone, tetrahydrofuran, room temperature; (ii) *t*-butyl (2S)-2-hydroxy-3-methylbutanoate, diisopropyl-azodicarboxylate, PPh_3_, room temperature; (iii) trifluoroacetic acid, CH_2_Cl_2_, 0°C, 5 h.

The inactive version of ARP101, the carboxylic acid, was synthesized according to the published procedure (62). Briefly, commercially available 4-phenylbenzene-1-sulfonyl chloride 2 was condensed with *O*-isopropylhydroxylamine hydrochloride to give the *O*-isopropyloxysulfonamide 3. This was then condensed with *t*-butyl (2S)-2-hydroxy-3-methylbutanoate via the Mitsunobu reaction to give the *t*-butyl ester 4, which was subsequently treated with trifluoroacetic acid to provide the inactive ARP101 analog 1 (Fig. 11).

### Viruses.

The following HCMV strains were used: a recombinant pp28-luciferase HCMV-Towne strain, which expresses luciferase under the control of the late HCMV gene promoter, pp28, and a pp28-luciferase GCV-resistant HCMV that contains a C607Y mutation in UL97 were used in luciferase assays (63, 64). The pp28-luciferase virus achieves a wide dynamic range of luciferase expression (6 to 7 logs) and highly correlates with the plaque assay. HCMV-Towne (ATCC VR-977) was used for immunofluorescence, immunoblotting, and immunoprecipitation assays. The HCMV-TB40 strain with a plasmid carrying the UL32 gene fused to green fluorescent protein (GFP) was obtained from ATCC (VR-1578) and used for plaque reduction assays.

### Cells and antiviral assays.

Human foreskin fibroblasts (HFFs), passage 8 to 16 (ATCC, CRL-2088), were grown in Dulbecco’s modified Eagle’s medium (DMEM) containing 10% fetal bovine serum (FBS) (Gibco, Carlsbad, CA) in a 5% CO_2_ incubator at 37°C. Plaque assays were performed with HCMV-TB40. HFFs were seeded at 1.5 million cells/plate in a 12-well plate and infected 24 h later at 200 PFU/well. Following virus adsorption (90 min), the virus was aspirated, and DMEM containing 4% fetal bovine serum (FBS) with 0.5% carboxymethyl cellulose was added with the compounds at indicated concentrations into triplicate wells. After incubation at 37°C for 8 days, the overlay was removed, and plaques were counted after crystal violet staining.

### Luciferase assay.

A total of 1.5 million HFFs were infected in 96-well plates with pp28-luciferase HCMV-Towne (MOI of 1). Infected treated HFFs were collected at 72 hpi, and lysates were assayed for luciferase activity using a luciferase assay kit (Promega, Madison, WI) on a SpectraMax i3X (Molecular Devices, San Jose, CA).

### Cell Titer-Glo luminescent cell viability assay.

A total of 1.5 million HFFs were plated in a 96-well plate, and on the following day, cells were treated with different concentrations of ARP101 and incubated for 3 or 8 days. Cell viability was determined by a Cell Titer-Glo luminescent cell viability assay (Promega, Madison, WI) according to the manufacturer’s protocol.

### Viral load by quantitative real-time PCR.

Total DNA was isolated from noninfected and HCMV-infected treated HFFs (MOI of 0.1 PFU/cell) using the Wizard SV genomic DNA isolation kit (Promega, Madison, WI). A US17 real-time PCR assay that targets 151 bp from the highly conserved US17 region of the HCMV genome was used (65). The primers and probe used for US17 were as follows: forward (F), 5′-GCGTGCTTTTTAGCCTCTGCA-3′; reverse (R), 5′-AAAAGTTTGTGCCCCAACGGTA-3′; and US17 probe, 6-carboxyfluorescein (FAM)–5′-TGATCGGGCGTTATCGCGTTCT-3′.

### Indirect immunofluorescence assay for HCMV entry.

ARP101 and GCV were diluted in serum-free medium and added to HFFs seeded on chamber slides 24 h before infection. After infection for 90 min (MOI of 1 PFU/cell) and washing with phosphate-buffered saline (PBS), cells were fixed, permeabilized, and air dried. Cells were incubated with mouse monoclonal anti-pp65 antibody at 37°C in humidified chambers for 1 h, washed three times with 0.1% Tween 20 in PBS (PBST), incubated with fluorescein isothiocyanate (FITC)-conjugated anti-mouse IgG (Sigma-Aldrich, St. Louis, MO) at 37°C in humidified chambers for 1 h, and washed with PBST (0.1% Tween 20). A drop of mount oil containing DAPI (4′,6-diamidino-2-phenylindole) (Santa Cruz Inc., Dallas, TX) was added to the slides before visualization with a Nikon Eclipse E-800 fluorescence microscope.

### SDS-PAGE and immunoblotting.

Whole-cell lysates (noninfected and infected with HCMV-Towne; MOI of 1 PFU/cell) were quantified for protein content with a bicinchoninic acid (BCA) protein assay kit (Pierce Chemical, Rockford, IL). Equivalent amounts of proteins were used for immunoblot analysis. Protein bands were visualized by chemiluminescence using a Western blotting luminol reagent (Thermo Scientific, Rockford, IL). Antibodies for detection of HCMV proteins were as follows: mouse monoclonal anti-pp65 (Vector Laboratories, Burlingame, CA), mouse anti-IE1 and -IE2 (MAb810; Millipore, Billerica, MA), mouse monoclonal anti-UL84 (Santa Cruz Biotechnology, Dallas, TX), and CMV pp28 (Virusys Corporation, Taneytown, MD). Other antibodies and related reagents were as follows: mouse monoclonal anti-β-actin (Millipore, Billerica, MA); rabbit polyclonal anti-LC3-II (Novus Biologicals, LLC, Littleton, CO); anti-SQSTM1/p62, ATG5, mTOR, p-mTOR, p-4EBP1, p-SQSTM1/p62 (Ser349), p-SQSTM1/p62 (S403), and Keap1 antibodies (Cell Signaling Technology, Beverly, MA); rabbit polyclonal anti-HO-1 (Enzo Life Sciences, Farmingdale, NY); rabbit polyclonal anti-Nrf2 (Invitrogen, Rockford, IL); horseradish peroxidase (HRP)-conjugated goat anti-rabbit IgG (Cell Signaling Technology); and HRP-conjugated sheep anti-mouse IgG (GE Healthcare, Waukesha, WI). The relative band intensities (normalized to respective internal controls) were analyzed by Bio-Rad Image Lab software and are mentioned under the respective blots.

### Lentivirus-mediated knockdown (KD) of ATG5 and Nrf2.

KD of ATG5 was conducted as described previously (26). Two Nrf2 short hairpin RNA (shRNA) plasmids (tet_pLKO.1_puro_shNRF2#1 and tet_pLKO.1_puro_shNRF2#2) were donated by Kevin Janes (Addgene plasmid no. 136584 and 136585). The corresponding control plasmid tet-pLKO.1_puro was donated by Dmitri Wiederschain (Addgene plasmid no. 21915). Individual shRNA constructs were packaged, and lentivirus particles containing shRNA were transduced into HFFs, followed by puromycin selection (2 μg/mL). Transduced cells were cultured for at least three passages before analysis of Nrf2 at the protein level. Control and Nrf2 KD HFFs were counted, and equal numbers of cells were plated before infection or treatment. Nrf2 KD #2 was confirmed by immunoblotting and used for all experiments involved.

### Coimmunoprecipitation and immunoblotting.

Two million HFFs were plated in a 6-well plate, infected with HCMV-Towne (MOI of 1), and treated with ARP101 (10 μM) for 48 and 72 h. Cells were harvested at both time points and lysed with an IP buffer containing 150 mM NaCl, 50 mM Tris, pH 7.5, 2 mM EDTA, 0.5% Triton X-100, and 0.5% NP-40. The lysates were sonicated on ice 3 times with pulse and rest for 10 s each. One milligram of lysate was precleared with bead slurry for 30 min. The precleared lysates were incubated with rabbit monoclonal anti-SQSTM/p62 (1:100; catalog no. 8025S; Cell Signaling Technology) or anti-Nrf2 antibody overnight on an end-end rotator. The antibody complexes were isolated using protein A/G beads (Santa Cruz Inc.) and washed three times with 50% IP buffer. The immunoprecipitated protein A/G beads were boiled in SDS sample buffer, and the supernatant was analyzed by SDS-PAGE after immunoblotting for p62, p-p62 (S349), p-p62 (S403), Keap1, Nrf2, HCMV pp65, and pp28, respectively, as previously described. Mouse monoclonal Keap1 antibody (sc-365626; Santa Cruz Biotechnology) was used for Keap1 detection. One percent of the cell lysate used for IP was loaded into the gel as “input.” Isotype control antibodies included polyclonal rabbit IgG (Abcam, Cambridge, MA). For immunoprecipitation of p-p62 (Ser349) (1:50; catalog no. ab2113224; Abcam, Waltham, MA), cells were infected, treated with ARP101 (10 μM) or in combination with K67 for 72 h, and lysed in radioimmunoprecipitation assay (RIPA) buffer (Thermo Scientific; catalog no. 89900) followed by sonication. The pulldown was analyzed by SDS-PAGE after immunoblotting for p-p62 (Ser349), Keap1, and LC3 II.

### Cellular fractionation.

Two million HFFs were plated on a 6-well plate. The following day, cells were infected with HCMV-Towne and treated with ARP101. At 48 and 72 h, cells were fractionated using the Qproteome Cell Compartment kit (Qiagen, Hilden, Germany) following the manufacturer’s instructions. Briefly, cells were washed and treated with specific buffers to obtain the cytoplasmic and nuclear fractions, precipitated with acetone, and quantified using a BCA assay kit (Thermo Scientific, Rockford, IL). An equal amount of protein was analyzed by immunoblotting, and cytoplasmic and nuclear fractions were confirmed by probing for GAPDH (Santa Cruz Inc.) and histone H3 (Cell Signaling Technology) as internal controls, respectively.

### ChIP assay.

Four million HFFs were used for each condition of the chromatin immunoprecipitation (ChIP) assay, using the Pierce magnetic ChIP kit (catalog no. 26157; Thermo Scientific). Briefly, HFFs were infected with HCMV-Towne (MOI of 1) and treated with active or inactive ARP101 (10 μM). At 48 and 72 hpi, cells were fixed with 1% paraformaldehyde, neutralized with 0.125 M glycine solution, washed, and harvested by scraping. The cell pellets were lysed using membrane extraction buffer, digested with micrococcal nuclease (MNase), and sonicated, and IP was performed with Nrf2 antibody (1:50; catalog no. ab62352; Abcam) and rabbit polyclonal IgG as negative control. Following IP, samples were eluted, DNA was recovered, and quantitative reverse transcriptase PCR (qRT-PCR) was performed using primer sets specific for the following promoter regions: *HMOX1* (forward [F], 5′-CCATCTGGCGCCGCTCTGC-3′; R, 5′-GAGCAGCTGGAACTCTGAGGA-3′), *SQSTM1/p62* (F, 5′-CTCTCAGGCGCCTGGGCTGCTGAG-3′; R, 5′-CGGCGGTGGAGAGTGGAAAATGCC-3′), *NQO1* (F, 5′-GCAGTCACAGTGACTCAGC-3′; R, 5′-TGTGCCCTGAGGTGCAA-3′), and *GCLC* (F, 5′-ATCGACTGCGGCAATCCTAG-3′; R, 5′-CGTGACTCAGCGCTTTGTG-3′). Normalized fold enrichment in each case was calculated using the formula (primer efficiency)^−ΔΔ^*^CT^* where ΔΔ*C_T_* = Δ*C_T_* [Nrf2] − Δ*C_T_* [IgG control]. *C_T_* stands for threshold cycle.

### RNA isolation and real-time qRT-PCR.

Total RNA was isolated from cultured cells using the RNeasy minikit (Qiagen). The RevertAid first-strand cDNA synthesis kit (Thermo Fisher Scientific, Waltham, MA) was used to synthesize first-strand cDNA from total RNA using oligo(dT) primers. Negative RT reaction mixtures were included to ensure the specificity of quantitative reverse transcriptase PCRs (qRT-PCRs). Synthesis of the first strand of cDNA from the mRNA template was carried out at 42°C for 1 h. qRT-PCR was performed in triplicates using primers specific for *GCLC* (F, 5′-GGATTTGGAAATGGGCAATTG-3′; R, 5′-CTCAGATATACTGCAGGCTTGGAA-3′), *HMOX-1* (F, 5′-ATGGCCTCCCTGTACCACATC-3′; R, 5′-TGTTGCGCTCAATCTCCTCCT-3′), *SQSTM1/p62* (F, 5′-CTGCCCAGACTACGACTTGTGT-3′; R, 5′-TCAACTTCAATGCCCAGAGG-3′), and *NQO1* (F, 5′-CGCAGACCTTGTGATATTCCAG-3′; R, 5′-CGTTTCTTCCATCCTTCCAGG-3′). GAPDH (F, 5′-CGGAGTCAACGGATTTGGTCGTAT-3′; R, 5′-AGCCTTCTCCATGGTGGTGAAGAC-3′) was used as the internal control. Viral pp65 (F, 5′-GCAGCCACGGGATCGTACT-3′; R, 5′-GGCTTTTACCTCACACGAGCATT-3′), IE1 (F, 5′-CTTAATACAAGCCATCCACA-3′; R, 5′-TAGATAAGGTTCATGAGCCT-3′), and IE2 (F, 5′-GCACACCCAACGTGCAGACTCGGC-3′; R, 5′-TGGCTGCCTCGATGGCCAGGCTC-3′) were used to track HCMV infection and the effect of the compounds on viral replication. Real-time PCR was performed on a Bio-Rad CFX Connect system (Bio-Rad, Hercules, CA).

### Cycloheximide chase assay.

HFFs were plated at 2 million cells/plate on 6-well plates, infected with HCMV-Towne (MOI of 1), and either left untreated or treated with ARP101 (10 μM). At 72 hpi, cells were treated with cycloheximide (100 μg/mL) to halt new protein synthesis. Cell lysates were collected for analysis of Keap1. Western blot (WB) analysis was performed at 1 h, 3 h, 5 h, 7 h, 9 h, and 12 h after cycloheximide treatment. β-Actin was probed as a loading control.

### Viral titration from supernatants by plaque assay.

Nrf2 KD and pLKO.1-Puro control HFFs were infected (HCMV-TB40, MOI of 1) and treated with different concentrations of ARP101 for 120 h. Supernatants were harvested and serially diluted in 1× DMEM to infect new HFFs (1 × 10^6^ cells in a 24-well plate). The infected cells were layered with 4% DMEM and carboxymethyl cellulose. The plates were stained with crystal violet at 8 days postinfection, and viral titer in PFU per milliliter was calculated based on the enumeration of plaque numbers.

### Statistical analysis.

The EC_50_ and CC_50_ values were calculated using GraphPad Prism software using the nonlinear curve fitting and the exponential form of the median effect equation, where percent inhibition = 1/[1 + (CC_50_ or EC_50_/drug concentration)*m*], where *m* reflects the slope of the concentration-response curve. All statistical analyses were performed using the GraphPad Prism software; individual points represent mean ± standard deviation (SD) (*n* ≥ 3). A single-factor analysis of variance (ANOVA) was used to determine the significance of comparing groups, and * represents *P* ≤ 0.05, ** represents *P* ≤ 0.01, *** represents *P* ≤ 0.001, and “ns” represents nonsignificant.
